# Factors affecting accuracy and precision in ultrasound guided radiotherapy

**DOI:** 10.1016/j.phro.2021.05.003

**Published:** 2021-05-29

**Authors:** Alexander Grimwood, Karen Thomas, Sally Kember, Georgina Aldis, Rebekah Lawes, Beverley Brigden, Jane Francis, Emer Henegan, Melanie Kerner, Louise Delacroix, Alexandra Gordon, Alison Tree, Emma J. Harris, Helen A. McNair

**Affiliations:** a Department of Radiotherapy, Royal Marsden NHS Foundation Trust and Institute of Cancer Research, Radiotherapy Department, Royal Marsden NHS Foundation Trust, Sutton SM2 5PT, United Kingdom; b Joint Department of Physics, Royal Marsden NHS Foundation Trust and Institute of Cancer Research, Radiotherapy Department, Royal Marsden NHS Foundation Trust, Sutton SM2 5PT, United Kingdom; c Department of Statistics and Computing, Royal Marsden NHS Foundation Trust and Institute of Cancer Research, Radiotherapy Department, Royal Marsden NHS Foundation Trust, Sutton SM2 5PT, United Kingdom

**Keywords:** Prostate cancer, Inter observer error, Ultrasound, Treatment margins, Image guidance

## Abstract

**Background and purpose:**

Transperineal ultrasound (TPUS) is used clinically for directly assessing prostate motion. Factors affecting accuracy and precision in TPUS motion estimation must be assessed to realise its full potential.

**Methods and materials:**

Patients were imaged using volumetric TPUS during the Clarity-Pro trial (NCT02388308). Prostate motion was measured online at patient set-up and offline by experienced observers. Cone beam CT with markers was used as a comparator and observer performance was also quantified. The influence of different clinical factors was examined to establish specific recommendations towards efficacious ultrasound guided radiotherapy.

**Results:**

From 330 fractions in 22 patients, offline observer random errors were 1.5 mm, 1.3 mm, 1.9 mm (left–right, superior-inferior, anteroposterior respectively). Errors increased in fractions exhibiting poor image quality to 3.3 mm, 3.3 mm and 6.8 mm. Poor image quality was associated with inconsistent probe placement, large anatomical changes and unfavourable imaging conditions within the patient. Online matching exhibited increased observer errors of: 3.2 mm, 2.9 mm and 4.7 mm. Four patients exhibited large systematic residual errors, of which three had poor quality images. Patient habitus showed no correlation with observer error, residual error, or image quality.

**Conclusions:**

TPUS offers the unique potential to directly assess inter- and intra-fraction motion on conventional linacs. Inconsistent image quality, inexperienced operators and the pressures of the clinical environment may degrade precision and accuracy. Experienced operators are essential and cross-centre standards for training and QA should be established that build upon current guidance. Greater use of automation technologies may further minimise uncertainties.

## 1 Introduction

Image guided radiotherapy (IGRT) is essential for mitigating interfraction motion during external beam radiotherapy for prostate cancer [1], [2]. The current standard of care for IGRT is cone beam CT (CBCT), often incorporating implanted intraprostatic fiducial markers to improve prostate localisation [3], [4], [5], [6]. Implanting markers is, however, an invasive technique that requires additional hospital resources and is not possible for all patients [7], [8].

Recent evidence demonstrates the efficacy of hypofractionated and stereotactic IGRT, demanding increased accuracy for both interfraction and intrafraction verification [9], [10], [11], [12], [13]. Clinical adoption of hypofractionation is increasingly common, driven in part by the COVID-19 pandemic [14].

Ultrasound (US) is a portable non-invasive, non-ionising and cost-efficient imaging solution that does not require implanted markers and is compatible with conventional C-arm linear accelerators for prostate position verification. Widespread adoption has been hampered by issues such as: operator training, prostate displacement from abdominal probe pressure, and inadequate tools for registering US with reference CT scans [11], [15], [16], [17]. Volumetric transperineal ultrasound (TPUS) ensures no treatment beam obstruction, reduces prostate motion due to inconsistent probe pressure and avoids reliance on bladder filling to achieve a suitable acoustic window. Commercial TPUS systems combine intrafraction motion monitoring with interfraction verification [18], [19], [20], [21], potentially reducing the need for CBCT or marker implantation. To realise this potential, factors affecting TPUS IGRT accuracy must be systematically examined to inform future guidance and establish best clinical practice.

Consensus regarding quantification of clinical factors affecting match accuracy is often lacking [22], [23], [24]. Factors include age and bladder volume, where differences >5 mm between TPUS and CBCT were reported in over 20% of 19 post-prostatectomy patients [25].

Reported interobserver variability (IOV) was reported as comparable to soft tissue localisation using CBCT [26], with differences between experienced observers varying by up to 19 mm [27]. A study involving seven radiation therapists also reported significant IOV improvements with observer experience [28]. Image quality optimisation and probe positioning are both operator dependent, being closely associated with training, IOV and overall accuracy [28], [29], [30]. Even so, no published consensus exists regarding TPUS image quality criteria, or probe positioning criteria.

QA recommendations have been published to limit systematic uncertainties, however recommendations regarding patient selection, systems integration, image interpretation and training requirements are less specific [30]. This study aimed to quantify clinical factors affecting TPUS accuracy and precision for interfraction matching. Specifically, we investigated how uncertainties are influenced by the clinical environment, image quality, patient habitus and prostate rotation. TPUS was compared to CBCT-CT matching to quantify uncertainties and present recommendations that complement current guidance.

## 2 Material and methods

### 2.1 Patient recruitment and staff training

Patients referred for radical radiotherapy to the prostate or prostate and seminal vesicles were consented for the Clarity-Pro trial (NCT02388308) approved by the Surrey and SE Coast Regional Ethics Committee, UK. Three gold fiducial markers, 1 mm diameter × 3 mm length, were implanted into the prostate one week prior to planning CT according to departmental protocol. Patients had no contraindications for marker insertion. Standard CBCT with fiducial IGRT and verification was used for all patients.

Five radiographers undertook in-house training in 2015 covering TPUS demonstrations, practical sessions, lectures and assessments. A further 21 radiographers completed the program between 2016 and 2017, all of whom participated in online TPUS data collection.

### 2.2 Treatment planning, delivery and quality assurance

Routine quality checks ensured a ±1 mm TPUS tracking tolerance. Recalibrations were performed weekly and when QC failures occurred.

An Elekta Clarity Autoscan TPUS system (Elekta AB, Sweden) was used. TPUS image and probe position was manually optimised using real-time 2D scanning, centred on the prostate with rectum, symphysis pubis and penile bulb also in view. A 3D reference image was acquired (US-Sim), followed by CT (1.25 mm slices) with minimal time between scans. The protocol changed for the last seven patients, adding another 3D TPUS scan immediately after CT to monitor patient motion.

Five field IMRT treatments delivered in twenty 3 Gy fractions were planned as standard of care (Pinnacle, Philips medical systems USA). CT and US-Sim volumes were fused, manually checked and a TPUS planning reference volume (PRV) created for interfraction registration. The PRV defined prostate on US-Sim within the planning target volume (PTV). Hyperechoic regions were included where possible to assist registration.

Online CBCT and TPUS image registrations were performed at treatment by a trained radiographer. After patient set-up, TPUS (US-Guide) and CBCT scans were acquired simultaneously by separate radiographers. CBCT-CT prostate matches comprised a bony anatomy registration, followed by fiducial registrations (XVI Synergy v5.1). Independently, the TPUS match comprised manually registering the US-Sim PRV to US-Guide. Due to staff rotation, patients were not always matched by the same radiographer. TPUS couch shifts were recorded and intrafraction monitoring initiated [31]. CBCT-CT matches determined clinical couch shifts. TPUS registration was conducted within the time required for CBCT matching to minimise disruption to treatment.

Offline TPUS registration was performed by three experienced observers (EH, HMcN, AG) using Clarity Review Software independently from the radiographers’ online matches. All images were inspected for hyperechoic features likely to be markers, as determined by the three observers following previously reported procedure [18]. If a feature was <3 mm from a fiducial marker on co-registered CT, markers were deemed visible in TPUS and capable of biasing registration accuracy. Patients with visible markers were thus excluded from this study.

### 2.3 Analysis

Residual errors, $E_{\mathrm{SM}}$, were calculated as the difference between TPUS and CBCT-CT couch shifts, S, for each fraction: $E_{\mathrm{SM}}=S_{\mathrm{TPUS}}-S_{\mathrm{CBCT}}$. Observer errors, $E_{\mathrm{OB}}$, were calculated for a given fraction as the difference between an observer’s TPUS shift, $S_{\mathrm{TPUS}}$, and a gold standard TPUS shift (TPUS-GS). The offline observer mean was used to calculate TPUS-GS: $S_{\mathrm{GS}}=\left(S_{TPUS1}+S_{TPUS2}+S_{TPUS3}\right)/3$. Observer errors were calculated for offline and online matches relative to TPUS-GS shifts: $E_{\mathrm{OB}}=S_{\mathrm{TPUS}}-S_{\mathrm{GS}}$.

Following the van Herk model [32], both residual and observer errors were estimated. Mean error, ε , was calculated in each patient. Systematic error, Σ, was the standard deviation between patients (eq 1–2). Error standard deviation in each patient, $\sigma_{p}$, was used to calculate random error, σ (Eq. (3)):

(1) $$\varepsilon =\frac{1}{n\cdot m}\overset{n\cdot m}{\underset{i=1}{\sum }}E_{i}$$

(2) $$\Sigma =\sqrt{\frac{1}{N}\overset{N}{\underset{i=1}{\sum }}{\left(\varepsilon -\overset{-}{\varepsilon }\right)}^{2}}$$

(3) $$\sigma =\sqrt{\frac{1}{N}\sum_{i=1}^{N}{{\sigma_{p}}_{i}}^{2}}$$

where N patients were matched by m observers over n fractions. Margins, T, required to account for all uncertainties were estimated [32]:

(4) $$T=2.5\sqrt{\Sigma^{2}}+0.7\sqrt{\sigma^{2}}$$

Previously reported intrafraction uncertainties, CBCT match errors and margins were tabulated against TPUS results for comparison [31], [33], [34].

### 2.4 Residual errors

Residual error ($E_{\mathrm{SM}}$) means and 95% limits of agreement were calculated. Correlations between CBCT marker couch shifts and TPUS were calculated using Pearson’s correlation coefficient. The $E_{\mathrm{SM}}$ interquartile range for each patient was ${IQR}_{p}$. Patients with no overlap between ${IQR}_{p}$ and the cohort IQR were considered to exhibit systematic errors, because the offset indicates a significant difference between average patient error from the population average. These cases were qualitatively examined for possible causes. Online and offline TPUS matches were compared to CBCT using Wilcoxon rank-sum tests.

### 2.5 Observer errors and environment

Mean observer errors and 95% limits of agreement were calculated. Systematic and random uncertainties were estimated ($E_{\mathrm{OB}}$). The compound effect of staff turnover and time pressure when having to complete online TPUS matches was investigated by a comparison to offline match results from three experts. Offline and online $E_{\mathrm{OB}}$ was compared using paired Wilcoxon rank-sum tests. Interobserver agreement was quantified using Pearson’s correlation coefficient and by calculating interobserver variation (IOV) [26]:

(5) $$\mathrm{RMS}=\sqrt{\frac{1}{n}\sum_{i=0}^{n}{{\sigma_{\mathrm{obs}}}_{i}}^{2}}$$

(6) $$\mathrm{IOV}=\sqrt{\frac{1}{N}\sum_{i=0}^{N}{{\mathrm{RMS}}_{i}}^{2}}$$

where RMS is the root mean square of the interobserver match variance, ${\sigma_{\mathrm{obs}}}^{2}$, for n fractions in each patient, and IOV is the root mean square of this value across N patients.

The effects of the online environment was estimated from a quadrature approximation of the respective $E_{\mathrm{OB}}$ variances (${\sigma_{\mathrm{OB}}}^{2}$):

(7) $${\sigma_{\mathrm{env}}}^{2}={\sigma_{\mathrm{OBonline}}}^{2}-{\sigma_{\mathrm{OBoffline}}}^{2}$$

### 2.6 Image quality

Sim-Guide TPUS pairs containing images of poor quality were identified by their consistently low interobserver agreement. Fractions with offline observer errors ($E_{\mathrm{OB}}$) greater than two standard deviations, $2\sigma_{\mathrm{OB}}$, in any direction were identified. Observers rematched these fractions, blinded to the magnitude and direction of the original errors. Fractions where $E_{\mathrm{OB}}$ remained beyond $2\sigma_{\mathrm{OB}}$ were considered poor quality. When rematching, observers annotated the TPUS-Sim and TPUS-Guide scans. A qualitative review of annotated rematch scans with still low interobserver agreement characterized common sources of poor image quality. Error distributions were compared before and after rematching using paired Wilcoxon rank-sum tests.

### 2.7 Prostate rotations

CBCT image registration uses six degrees of freedom (i.e. rotations and translations) whilst Clarity only considers translation. The relationship between rotations measured on CBCT and TPUS-GS error was explored using linear regression and Pearson correlation coefficient.

### 2.8 Patient habitus and comfort

The relationship between patient body mass index (BMI) and mean TPUS-GS error was investigated using linear regression. Transperineal distance from prostate apex to probe surface was recorded on TPUS images and its relationship to both BMI and image quality examined using Pearson correlation. Finally, patients were asked to rate comfort and ease of positioning using a 4-point Likert scale.

## 3 Results

### 3.1 Patient recruitment

A total of 42 patients were recruited. Of these 22 had no visible fiducial markers on ultrasound and were included for analysis and labelled alphabetically. 21 patients were treated with 20 fractions and one patient treated with 19 fractions. Of the 439 fractions delivered, 341 had both CBCT and TPUS couch shifts available. The missing image pairs were the result of the patient being treated on a non-TPUS linac, time constraints, or TPUS availability due to servicing or deployment elsewhere. In these cases, a dummy probe was used to replicate patient position.

Eleven fractions (n=11) were identified for exclusion from the analysis during offline registration for of the following reasons:

(i) The prostate was mostly outside the TPUS field of view (n=2).
(ii) A noticeable systematic shift for a single patient between CBCT and TPUS after three fractions required realignment between reference TPUS and CT. Retrospective examination of the CT-TPUS fusion showed the prostate had moved posterior between planning CT and TPUS acquisitions (n=3).
(iii) Acquisition software upgrade between treatments meant comparable TPUS image pairs were unavailable (n=6).

**Fig. 1:**
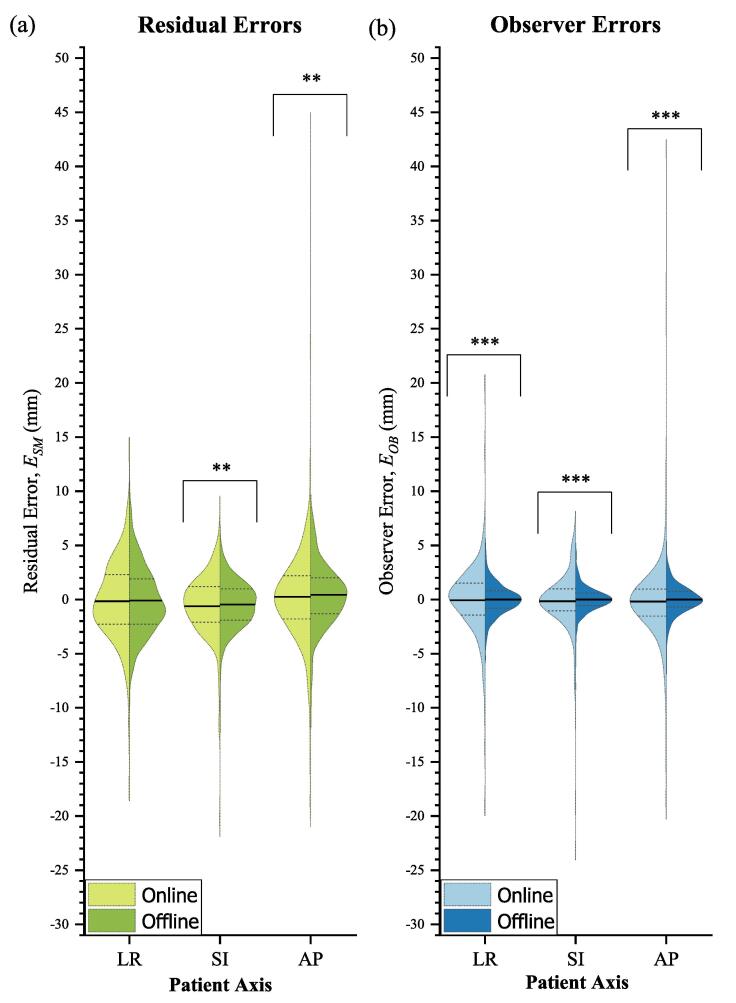
Online and offline residual error distributions (a) and observer error distributions (b) for left–right (LR), superior-inferior (SI) and anteroposterior (AP) patient axes.

**Fig. 2:**
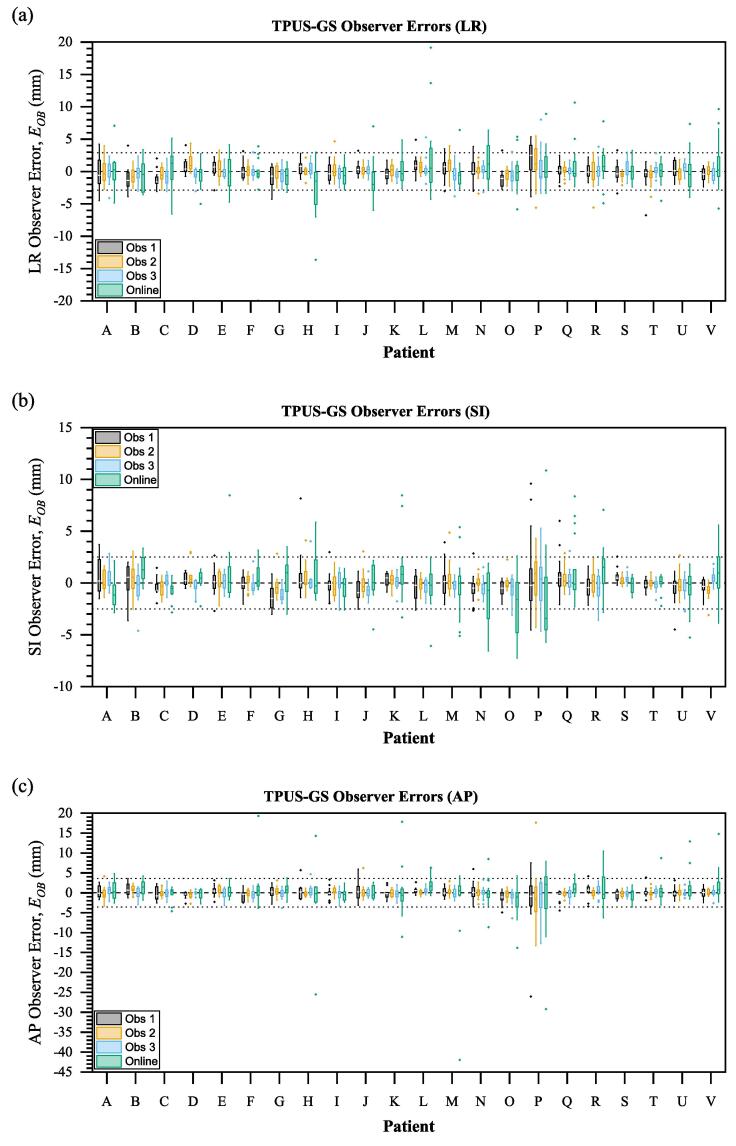
Individual offline and online observer error distributions for all 22 patients in (a) left–right, (b) superior-inferior and (c) anteroposterior axes. Dashed lines indicate mean offline error and dotted lines indicate 95% limits of agreement*.*

**Fig. 3:**
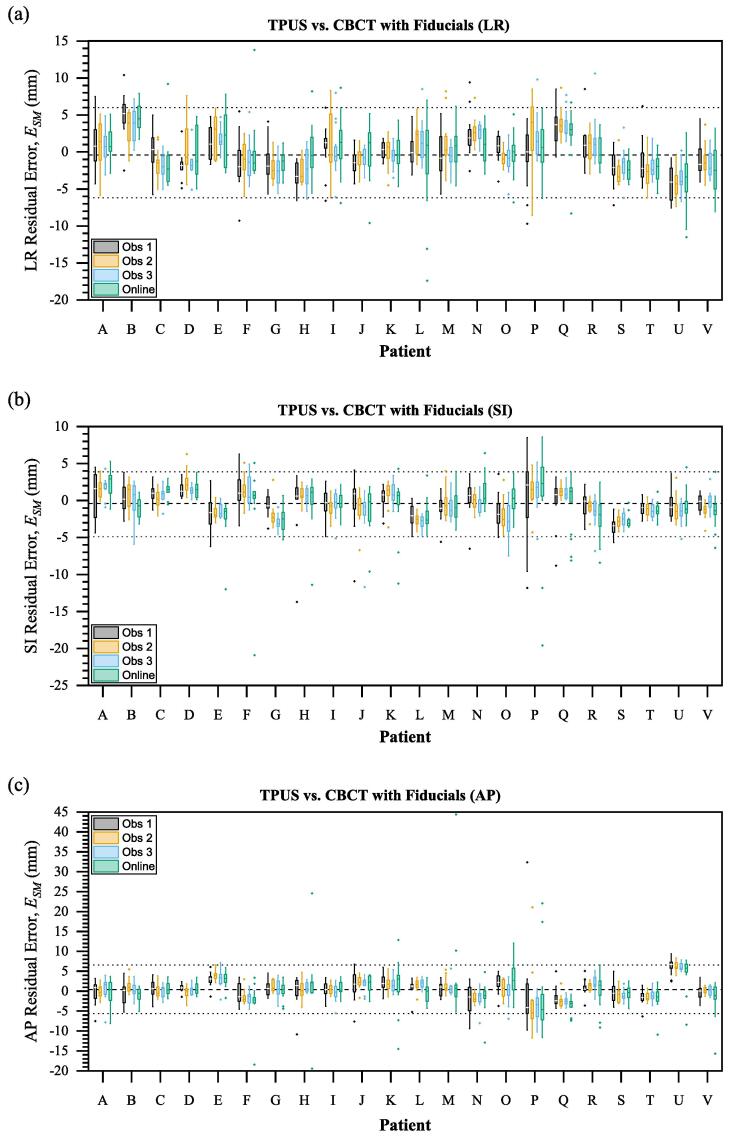
Individual offline and online residual error distributions for all 22 patients in (a) left–right, (b) superior-inferior and (c) anteroposterior axes. Dashed lines indicate mean offline error and dotted lines indicate 95% limits of agreement*.*

The remaining image pairs from 330 fractions were used for analysis of observer and residual errors. Error distributions are shown in Fig. 1 (Fig. 2, Fig. 3 show patient-specific distributions).

### 3.2 Residual errors

**Fig. 4:**
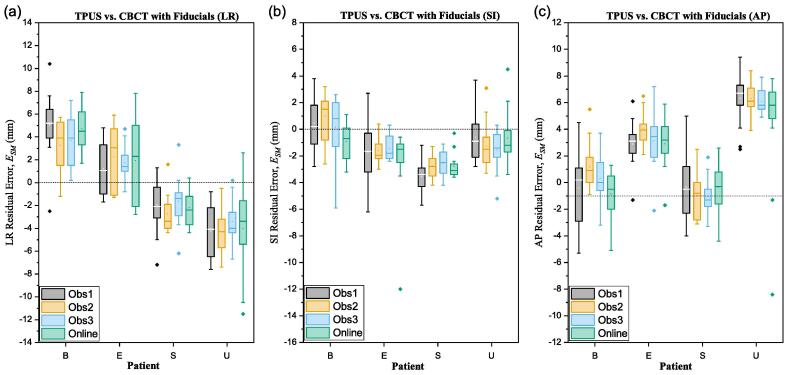
Residual error distributions for 4 patients (B, E, S, U) with significant systematic biases in at least one of: (a) left–right, (b) superior-inferior, or (c) anteroposterior patient axes.

TPUS was within 3 mm of CBCT ($E_{\mathrm{SM}}<3$ mm) for 62% of online and 68% of offline matches. For $E_{\mathrm{SM}}<5$ mm, agreement was 85% online and 88% offline. Mean (95% LOA) errors are listed in Table 1.CBCT shifts exhibited strong Pearson correlation with TPUS matches both online (LR: 0.60, SI: 0.81, AP: 0.81) and offline (LR: 0.63 ± 0.06, SI: 0.86 ± 0.05, 0.86 ± 0.05). Mean online residual errors ($E_{\mathrm{SM}}$) were significantly different to offline in two axes (LR: p=0.11, SI: p=0.01, AP: p=0.01) (Fig. 1). The estimated systematic and random residual errors are shown in Table 2. TPUS matches from 4 patients exhibited large systematic errors compared to CBCT, plotted in Fig. 4, equating to a group systematic error $\Sigma_{\mathrm{SM}}=$ 3.5 mm, 1.2 mm, 3.0 mm (LR, SI, AP respectively). All systematic errors except one were in the LR and AP axes. Patients B, E and S exhibited changes in the appearance of features between Sim and Guide scans, whilst patient U suffered consistently poor contrast with few discernible anatomical features within the prostate.

**Table 1:** Mean TPUS error values (mm) and 95% limits of agreement for observer (OB) and residual (SM) errors.

	Online			Offline		
	LR	SI	AP	LR	SI	AP
*OB*						
Mean $E_{\mathrm{OB}}$	0.1	0.2	0.2	0.0	0.0	0.0
95% LOA	−6.2	−5.5	−8.9	−2.9	−2.5	−3.6
	6.3	5.5	9.2	2.9	2.5	3.6

*SM*						
Mean $E_{\mathrm{SM}}$	−0.2	−0.6	0.2	−0.1	−0.5	0.4
95% LOA	−7.4	−6.9	−9.7	−6.2	−4.9	−5.6
	7.0	5.6	10.2	6.0	3.9	6.5

**Table 2:** Systematic (Σ) and random (σ) errors with Van Herk margins (T) in mm for online TPUS, offline TPUS and previously reported CBCT soft-tissue matches. Observer errors (OB). Residual errors (SM) assume CBCT fiducial matches as ground-truth. Previously reported intrafraction motion (IM) data are added to estimate a complete treatment margin (**T**).

	Online			Offline			CBCT (soft-tissue match)		
	LR	SI	AP	LR	SI	AP	LR	SI	AP
*OB*							Hirose et al., 2020 [33]		
Σ	1.1	1.0	1.2	0.0	0.0	0.0	0.5	0.9	0.9
σ	3.2	2.9	4.7	1.5	1.3	1.9	1.1	2.2	1.8
T	5.0	4.5	6.3	1.1	0.9	1.4	2.0	3.8	3.5
*SM*							Moseley et al. 2007 [31]		
Σ	1.9	1.4	2.1	2.0	1.3	1.9	0.6	2.1	2.0
σ	3.3	3.1	4.9	2.4	1.9	2.6	0.9	2.3	2.2
T	5.0	5.7	6.3	1.1	0.9	1.4	2.0	3.8	3.5
*IM*	Pang et al. [34]			Pang et al. [34]			Pang et al. [34]		
Σ	0.3	0.7	0.8	0.3	0.7	0.8	0.3	0.7	0.8
σ	0.8	1.1	1.3	0.8	1.1	1.3	0.8	1.1	1.3
T	1.2	2.6	2.8	1.2	2.6	2.8	1.2	2.6	2.8
T	8.7	7.7	11.0	7.2	5.6	7.5	3.5	8.3	8.0

### 3.3 Observer errors and environment

Mean (95% LOA) errors are listed in Table 1. Systematic and random observer errors for both online and offline matches are given in Table 2. Offline IOV was 1.5 mm, 1.2 mm, 1.5 mm (LR, SI, AP). Wilcoxon rank-sum testing of $E_{\mathrm{OB}}$ distributions (Fig. 1) indicated significant differences between online and offline (p<0.001 for all axes). The online environment was estimated to contribute to random observer errors $\sigma_{\mathrm{env}}=2.8$ mm, 2.6 mm, 4.3 mm (LR, SI and AP respectively). Median offline interobserver correlations were 0.80(±0.05), 0.88(±0.04), 0.90(±0.02) (LR, SI, AP).

### 3.4 Image quality

**Fig. 5:**
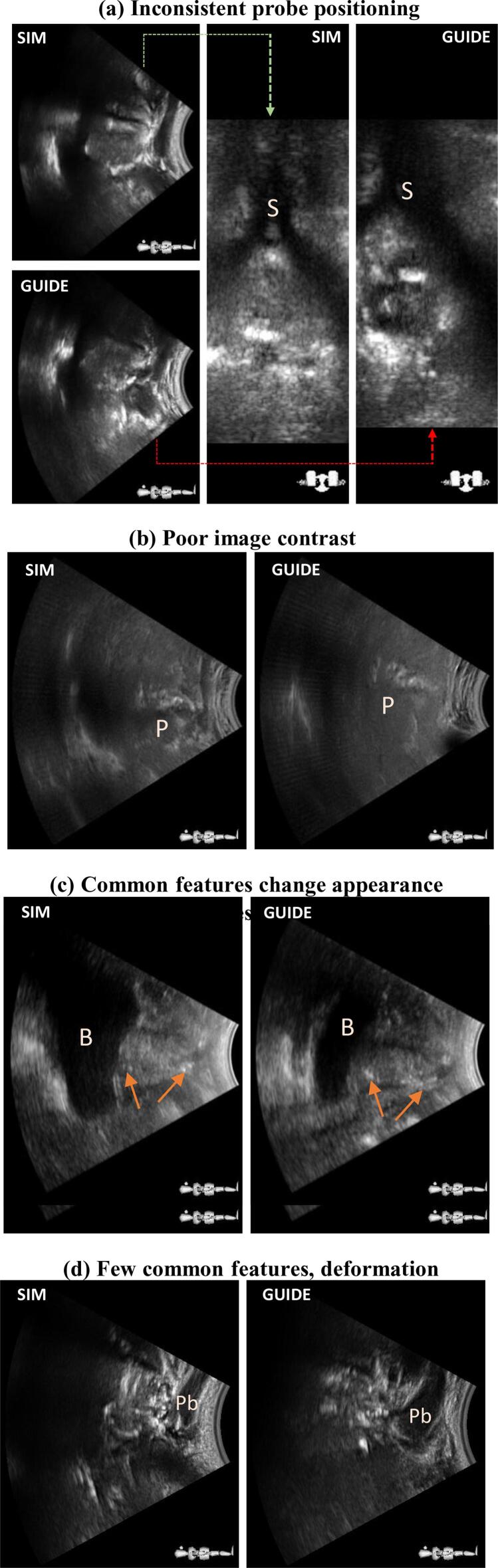
Three categorisations of poor image quality identified when reviewing fractions with high observer disagreement, or systematic offsets: (a) poor/inconsistent probe positioning between Sim and Guide, as evidenced by the offset pubic symphisys (S) position, leading to inconsistent appearance of anatomy; (b) poor intrinsic image contrast in patient P resulting in few discernible prostate (P) features; (c-d) inconsistent appearance of anatomical landmarks (arrows) due to internal changes, such as rectum or bladder filling and changes in probe pressure evidenced by penile bulb (Pb) size.

Of 75 fractions with an observer error $E_{\mathrm{OB}}>2\sigma_{\mathrm{OB}}$, 40 fractions among 17 patients exceeded this error threshold after rematching. Patient P accounted for 10 fractions and produced the highest mean random observer error ($\sigma_{\mathrm{OB}}$) at LR: 3.3 mm, SI: 3.3 mm, AP: 6.8 mm. This patient’s reference and treatment images showed poor contrast, with no discernible prostate features, or visible prostate capsule (Fig. 5b). Two patients (B and U) exhibiting large systematic errors were also identified as having poor image quality. Observer variance across the entire patient cohort was reduced after rematching from: 1.7 mm, 1.4 mm, 2.0 mm (LR, SI, AP respectively) to: 1.5 mm, 1.3 mm, 1.8 mm, but changes were not significant (Wilcoxon rank-sum p>0.1 in all axes). Sources of poor image quality were categorised and are summarised with examples in Fig. 5. The categories are:

a) poor probe placement resulting in the prostate appearing partially outside the field of view, or inconsistencies in appearance of anatomical structures between scans (Fig. 5a)
b) inadequate scan optimisation, probe coupling, or unfavourable imaging conditions (high tissue density, scarring, limited acoustic window) (Fig. 5b)
c) changes in bladder or rectal filling, posture, or probe pressure, producing significant changes in the position and appearance of anatomical structures (Fig. 5c and 5d)

### 3.5 Prostate rotations and patient habitus

Poor correlation between $E_{\mathrm{SM}}$, prostate rotation, patient BMI, or prostate apex depth was found. Pearson coefficients were between −0.2 and 0.2 in all directions for all comparisons. However, mean (SD) prostate apex depth was 33.7 (5.6) mm, with the largest observed in the same patient associated with consistently poor image quality. Linear regression testing found no significant relationship between any factor (p>0.05 in all cases).

### 3.6 Patient acceptability

All patients found positioning ‘moderately’ or ‘very easy’, agreeing the ‘probe was comfortable when positioned’. Only one patient rated maintaining position as ‘slightly easy’, with all others stating ‘moderately’ or ‘very easy’. The mean (SD) offline registration times for three observers was 81 (43) s, 171 (90) s and 212 (66) s.

## 4 Discussion

An investigation into the sources of TPUS match errors was conducted. Both systematic and random uncertainties were calculated between observers and against the clinical standard of care (CBCT with fiducial markers). From Table 2, online TPUS planning target volume (PTV) margins were estimated to be: 8.7 mm, 7.7 mm and 11.0 mm (left–right, superior-inferior, anteroposterior) around the clinical target volume (CTV). These estimates were not adequately conformal for advanced treatments, such as rectal sparing in moderately hypofractionated prostate radiotherapy, where typical posterior CTV to PTV margins are 3–7 mm (and 5–9 mm non-posterior) [35]. However, our TPUS estimates were inflated by measurement errors associated with CBCT marker matches and also ignored Clarity Autoscan’s intrafraction motion management capability. Actual TPUS margins are likely to be significantly smaller as a result. Sufficient TPUS conformity was achieved by experienced observers offline: ≤7.2 mm (non-anteroposterior) and ≤7.5 mm (anteroposterior) despite these inflated uncertainties (Table 2). Recommendations in this section describe ways to enable full TPUS guidance in future. However, TPUS monitoring may also be used in conjunction with CBCT positioning to capitalise on the accuracy of CBCT marker matching.

A review of individual Clarity scans with large match errors suggested poor, or inconsistent image quality contributed to match uncertainty. Consistently large systematic and random errors were observed in a single patient (P), which was attributed to poor innate imaging conditions, underscoring the need for enhanced patient selection. Systematic errors also arose from anatomical motion in the pause between TPUS and CT acquisitions (~90 s), possibly attributable to transitory rectal changes [36]. Furthermore, the few fractions that exhibited systematic errors with no obvious cause upon reviewing the images could indicate insufficient integration of Clarity with other clinical systems.

Offline uncertainties were significantly smaller than online across the entire cohort, likely as a result of reduced time pressures and greater observer experience. Residual offline errors were comparable to previously reported CBCT soft tissue matches, whereas online random uncertainties were larger. This result, in combination with a recorded increase in online observer errors, underlines the need to support and retain experienced staff in clinic. Poor image quality significantly reduced interobserver agreement, as exemplified by patient P exhibiting consistently low image quality with the highest offline observer error ($\sigma_{\mathrm{OB}}$) LR: 3.3 mm, SI: 3.3 mm, AP: 6.8 mm.

Larger online uncertainties may have been due to high staff turnover and competing clinical priorities. Sixteen radiographers were trained over two years, making it challenging to maintain sufficient clinical experience, as recommended by guidance (TG154) [30]. Experience that is essential for optimising probe position, image quality and for accurate prostate matching [29], [40]. Studies often report operators as being ‘well trained’ and this requirement has been cited as a barrier to implementation [26], [41]. Additionally, TPUS was not used for clinical IGRT decisions, meaning less priority was assigned online to achieving optimal results during our study.

Matches performed using CBCT with markers are regarded as the gold standard in prostate verification [31]. However, centres in the UK perceive the invasiveness of implantation and infection risks as barriers to implementation, making it imperative to assess alternatives [7], [8]. CBCT soft-tissue prostate matching is also extensively used, warranting comparison against TPUS.

*Recommendation 1*: Increase use of emerging technologies to automate and simplify probe positioning [37], [38]. These tools make US-IGRT systems easier to use, improves image quality and reduces both residual and observer errors.

*Recommendation 2:* Perform intermodality checks (e.g. CBCT vs. TPUS) for systematic biases over the first 3 fractions as described by Fargier-Voiron et al. [36]*.* Systematic errors could be detected early and corrected, significantly reducing TPUS match uncertainties.

*Recommendation 3*: QA schedules should be integrated with other clinical systems to mitigate systematic errors. Simultaneous intermodality checks are recommended between CT, CBCT and TPUS – similar to those routinely used for other multimodality systems, such as PET-CT [39].

*Recommendation 4*: Patient assessment of suitability for TPUS IGRT should be expanded to include an evaluation of innate image quality, possibly via a preliminary imaging session.

*Recommendation 5*: Image registration algorithms should be integrated into the matching workflow*.* Online observer errors could be significantly reduced, even among inexperienced users, and matching times reduced [27]. Further error reductions may be possible through contouring and registration of additional anatomical structures, such as the penile bulb, calcifications and inferior bladder wall.

*Recommendation 6*: Staff continuity is paramount*.* Radiographers should scan the same patient throughout the course of treatment where possible to improve online consistency in probe placement and image interpretation. Recognition and actioning of anatomical changes should be conducted as outlined in guidance (TG154) [30]. Planning structure delineation should incorporate both TPUS scans and experienced staff to improve detection of systematic errors.

Setup differences between TPUS and CBCT can vary substantially between patient axes, fractions, patients and studies. Disparate reporting methodologies limit cross-centre comparisons, creating a need to establish best practice and improve cross-centre harmonisation. Li et al. reported on 177 fractions across 7 patients, producing random residual uncertainties of 1.42 mm, 1.82 mm, 1.56 mm (LR, SI, AP), and systematic uncertainties of 1.15 mm, 1.10 mm, 0.90 mm (LR, SI, AP) [22]. The errors are smaller than those measured in our study, possibly due to their inclusion of patients with visible markers and our larger cohort size (330 fractions across 22 patients). Richter et al. reported a 10 patient, 150 fraction study with residual error 95% LOAs of LR: −5.0 mm, 8.0 mm; SI: −9.4 mm, 6.5 mm; AP: −7.1 mm, 8.2 mm [23] – larger than our offline errors and comparable to online measurements. By comparison Fargier-Voiron et al. used an intermodality correction and reported 95% LOAs of LR: −4.5 mm, 4.3 mm; SI: −8.3 mm, 4.5 mm; AP: −3.5 mm, 6.9 mm for a 427 fraction study across 13 patients [26]. Interobserver variation (IOV) was comparable in the same study: 1.9 mm, 1.1 mm, 1.3 mm (LR, SI, AP) [26]. Interobserver correlations reported by Pang et al. were also comparable: 0.68(±0.24), 0.91(±0.09), 0.96(±0.04) (LR, SI, AP) [28]. Camps et al. thoroughly reviewed reported results from different studies [42].

Prostate rotations, depth and patient BMI had no discernible effect on match errors. The distance between perineum and prostate was reported to affect image quality in earlier studies [24], however no relationship was identified in this study. Reduced image quality was observed in fractions where anatomical changes occurred between simulation and guide scans, resulting in larger match errors. Differences in prostate morphology over the full course of treatment were not assessed, but changes to volume and marker motion have been reported [43], [44]. Such changes could degrade agreement between CBCT and TPUS matches, underlining the need for better intermodality evaluation.

In conclusion, TPUS offers the unique potential to directly measure both interfraction motion and monitor intrafraction motion on conventional linacs. Inconsistent image quality, inexperienced operators and the pressures of the clinical environment significantly degrade both registration precision and accuracy. Experienced operators are essential and cross-centre standards for both training and QA should be established that build upon current guidance. Greater use of automation technologies is also required to further minimise uncertainties.

## Declaration of Competing Interest

The authors declare the following financial interests/personal relationships which may be considered as potential competing interests: Alison Tree reports support from Elekta, Varian and Accuray as a clinical research fellow working on other projects (not related to this project) and personally has received honoraria and travel grants from Elekta to cover meeting attendance. Emma Harris reports a non-financial research agreement with Elekta from 2014 until 2017 covering support and advice for the trial from which this study’s data was generated. A current research framework agreement between ICR and Elekta Ltd. is also reported, which does not pertain to this study. All other authors have no competing interests to declare.

## References

[1] Roeske J.C., Forman J.D., Mesina C.F., He T., Pelizzari C.A., Fontenla E. (1995). Evaluation of changes in the size and location of the prostate, seminal vesicles, bladder, and rectum during a course of external beam radiation therapy. Int J Radiat Oncol Biol Phys.

[2] Vigneault E., Pouliot J., Laverdière J., Roy J., Dorion M. (1997). Electronic portal imaging device detection of radioopaque markers for the evaluation of prostate position during megavoltage irradiation: a clinical study. Int J Radiat Oncol Biol Phys.

[3] Alasti H., Petric M.P., Catton C.N., Warde P.R. (2001). Portal imaging for evaluation of daily on-line setup errors and off-line organ motion during conformal irradiation of carcinoma of the prostate. Int J Radiat Oncol Biol Phys.

[4] Crook J.M., Raymond Y., Salhani D., Yang H., Esche B. (1995). Prostate motion during standard radiotherapy as assessed by fiducial markers. Radiother Oncol.

[5] McNair H.A., Hansen V.N., Parker C.C., Evans P.M., Norman A., Miles E. (2008). A comparison of the use of bony anatomy and internal markers for offline verification and an evaluation of the potential benefit of online and offline verification protocols for prostate radiotherapy. Int J Radiat Oncol Biol Phys.

[6] Wu J., Haycocks T., Alasti H., Ottewell G., Middlemiss N., Abdolell M. (2001). Positioning errors and prostate motion during conformal prostate radiotherapy using on-line isocentre set-up verification and implanted prostate markers. Radiother Oncol.

[7] Alexander S.E., Kinsella J., McNair H.A., Tree A.C. (2018). National survey of fiducial marker insertion for prostate image guided radiotherapy. Radiography.

[8] Gill S., Li J., Thomas J., Bressel M., Thursky K., Styles C. (2012). Patient-reported complications from fiducial marker implantation for prostate image-guided radiotherapy. Br J Radiol.

[9] Alayed Y., Quon H., Cheung P., Chu W., Chung H.T., Vesprini D. (2019). Two versus five stereotactic ablative radiotherapy treatments for localized prostate cancer: a quality of life analysis of two prospective clinical trials. Radiother Oncol.

[10] Dearnaley D., Syndikus I., Mossop H., Khoo V., Birtle A., Bloomfield D. (2016). Conventional versus hypofractionated high-dose intensity-modulated radiotherapy for prostate cancer: 5-year outcomes of the randomised, non-inferiority, phase 3 chhip trial. Lancet Oncol.

[11] Draulans C., De Roover R., van der Heide U.A., Haustermans K., Pos F., Smeenk R.J. (2019). Stereotactic body radiation therapy with optional focal lesion ablative microboost in prostate cancer: topical review and multicenter consensus. Radiother Oncol.

[12] Raziee H., Moraes F.Y., Murgic J., Chua M.L.K., Pintilie M., Chung P. (2017). Improved outcomes with dose escalation in localized prostate cancer treated with precision image-guided radiotherapy. Radiother Oncol.

[13] Widmark A., Gunnlaugsson A., Beckman L., Thellenberg-Karlsson C., Hoyer M., Lagerlund M. (2019). Ultra-hypofractionated versus conventionally fractionated radiotherapy for prostate cancer: 5-year outcomes of the hypo-rt-pc randomised, non-inferiority, phase 3 trial. Lancet.

[14] Zaorsky N.G., Yu J.B., McBride S.M., Dess R.T., Jackson W.C., Mahal B.A. (2020). Prostate cancer radiotherapy recommendations in response to covid-19. Adv Radiat Oncol.

[15] Ariyaratne H., Chesham H., Alonzi R. (2017). Image-guided radiotherapy for prostate cancer in the United Kingdom: a national survey. Br J Radiol.

[16] Fargier-Voiron M., Presles B., Pommier P., Rit S., Munoz A., Liebgott H. (2014). Impact of probe pressure variability on prostate localization for ultrasound-based image-guided radiotherapy. Radiother Oncol.

[17] McNair H.A., Mangar S.A., Coffey J., Shoulders B., Hansen V.N., Norman A. (2006). A comparison of ct- and ultrasound-based imaging to localize the prostate for external beam radiotherapy. Int J Radiat Oncol Biol Phys.

[18] Grimwood A., McNair H.A., O'Shea T.P., Gilroy S., Thomas K., Bamber J.C. (2018). In vivo validation of elekta's clarity autoscan for ultrasound-based intrafraction motion estimation of the prostate during radiation therapy. Int J Radiat Oncol Biol Phys.

[19] Richardson A.K., Jacobs P. (2017). Intrafraction monitoring of prostate motion during radiotherapy using the clarity((r)) autoscan transperineal ultrasound (tpus) system. Radiography.

[20] Bertholet J, Knopf A, Eiben B, McClelland J, Grimwood A, Harris E, et al. Real-time intrafraction motion monitoring in external beam radiotherapy. Phys Med Biol 2019;64:15TR01, doi: 10.1088/1361-6560/ab2ba8. 10.1088/1361-6560/ab2ba8 PMC7655120 31226704

[21] Sihono D.S.K., Ehmann M., Heitmann S., von Swietochowski S., Grimm M., Boda-Heggemann J. (2018). Determination of intrafraction prostate motion during external beam radiation therapy with a transperineal 4-dimensional ultrasound real-time tracking system. Int J Radiat Oncol Biol Phys.

[22] Li M., Ballhausen H., Hegemann N.-S., Reiner M., Tritschler S., Gratzke C. (2017). Comparison of prostate positioning guided by three-dimensional transperineal ultrasound and cone beam ctVergleich der Prostatapositionierung anhand dreidimensionalem transperinealem Ultraschall und Cone-beam-CT. Strahlenther Onkol.

[23] Richter A., Polat B., Lawrenz I., Weick S., Sauer O., Flentje M. (2016). Initial results for patient setup verification using transperineal ultrasound and cone beam ct in external beam radiation therapy of prostate cancer. Radiat Oncol.

[24] Trivedi A., Ashikaga T., Hard D., Archambault J., Lachaine M., Cooper D.T. (2017). Development of 3-dimensional transperineal ultrasound for image guided radiation therapy of the prostate: Early evaluations of feasibility and use for inter- and intrafractional prostate localization. Pract Radiat Oncol.

[25] Zhou S., Luo L., Li J., Lin M., Chen L.i., Shao J. (2019). Analyses of the factors influencing the accuracy of three-dimensional ultrasound in comparison with cone-beam ct in image-guided radiotherapy for prostate cancer with or without pelvic lymph node irradiation. Radiat Oncol.

[26] Fargier-Voiron M., Presles B., Pommier P., Munoz A., Rit S., Sarrut D. (2016). Evaluation of a new transperineal ultrasound probe for inter-fraction image-guidance for definitive and post-operative prostate cancer radiotherapy. Phys Med.

[27] Grimwood A., Rivaz H., Zhou H., McNair H.A., Jakubowski K., Bamber J.C. (2020). Improving 3d ultrasound prostate localisation in radiotherapy through increased automation of interfraction matching. Radiother Oncol.

[28] Pang E.P.P., Knight K., Baird M., Tuan J.K.L. (2017). Inter- and intra-observer variation of patient setup shifts derived using the 4d tpus clarity system for prostate radiotherapy. Biomed Phys Eng Express.

[29] Hilman S., Smith R., Masson S., Coomber H., Bahl A., Challapalli A. (2017). Implementation of a daily transperineal ultrasound system as image-guided radiotherapy for prostate cancer. Clin Oncol (R Coll Radiol).

[30] Molloy J.A., Chan G., Markovic A., McNeeley S., Pfeiffer D., Salter B. (2011). Quality assurance of u.S.-guided external beam radiotherapy for prostate cancer: report of aapm task group 154. Med Phys.

[31] Moseley D.J., White E.A., Wiltshire K.L., Rosewall T., Sharpe M.B., Siewerdsen J.H. (2007). Comparison of localization performance with implanted fiducial markers and cone-beam computed tomography for on-line image-guided radiotherapy of the prostate. Int J Radiat Oncol Biol Phys.

[32] VANHERK M. (2004). Errors and margins in radiotherapy. Semin Radiat Oncol.

[33] Hirose T.-A., Arimura H., Fukunaga J.-I., Ohga S., Yoshitake T., Shioyama Y. (2020). Observer uncertainties of soft tissue-based patient positioning in igrt. J Appl Clin Med Phys.

[34] Pang E.P.P., Knight K., Fan Q., Tan S.X.F., Ang K.W., Master Z. (2018). Analysis of intra-fraction prostate motion and derivation of duration-dependent margins for radiotherapy using real-time 4d ultrasound. Phys Imag Radiat Oncol.

[35] Brand D.H., Tree A.C., Ostler P., van der Voet H., Loblaw A., Chu W. (2019). Intensity-modulated fractionated radiotherapy versus stereotactic body radiotherapy for prostate cancer (pace-b): acute toxicity findings from an international, randomised, open-label, phase 3, non-inferiority trial. Lancet Oncol.

[36] Fargier-Voiron M., Presles B., Pommier P., Munoz A., Rit S., Sarrut D. (2015). Ultrasound versus cone-beam ct image-guided radiotherapy for prostate and post-prostatectomy pretreatment localization. Phys.

[37] Grimwood A., McNair H., Hu Y., Bonmati E., Barratt D., Harris E.J., Martel A.L., Abolmaesumi P., Stoyanov D., Mateus D., Zuluaga M.A., Zhou S.K., Racoceanu D., Joskowicz L. (2020). Assisted probe positioning for ultrasound guided radiotherapy using image sequence classification. Med image comput comput assist interv.

[38] Camps S.M., Houben T., Carneiro G., Edwards C., Antico M., Dunnhofer M. (2020). Automatic quality assessment of transperineal ultrasound images of the male pelvic region, using deep learning. Ultrasound Med Biol.

[39] Mawlawi OR, Jordan DW, Halama JR, Schmidtlein CR, Wooten WW, Aapm report no. 126: Pet/ct acceptance testing and quality assurance, 2019.

[40] Pang EPP, Knight K, Leung RW, Wang MLC, Chan JWS, Low GK, et al. Technical considerations for positioning and placement of a transperineal ultrasound probe during prostate radiotherapy. J Med Radiat Sci 2020, doi: 10.1002/jmrs.439. 10.1002/jmrs.439 PMC8168066 33017863

[41] McNair H.A., Hafeez S., Taylor H., Lalondrelle S., McDonald F., Hansen V.N. (2015). Radiographer-led plan selection for bladder cancer radiotherapy: Initiating a training programme and maintaining competency. Br J Radiol.

[42] Camps S.M., Fontanarosa D., de With P.H.N., Verhaegen F., Vanneste B.G.L. (2018). The use of ultrasound imaging in the external beam radiotherapy workflow of prostate cancer patients. Biomed Res Int.

[43] Kupelian P.A., Willoughby T.R., Meeks S.L., Forbes A., Wagner T., Maach M. (2005). Intraprostatic fiducials for localization of the prostate gland: monitoring intermarker distances during radiation therapy to test for marker stability. Int J Radiat Oncol Biol Phys.

[44] van der Heide U.A., Kotte A.N.T.J., Dehnad H., Hofman P., Lagenijk J.J.W., van Vulpen M. (2007). Analysis of fiducial marker-based position verification in the external beam radiotherapy of patients with prostate cancer. Radiother Oncol.

